# Maximizing Realism: Mapping Plastic Particles at the Ocean Surface Using Mixtures of Normal Distributions

**DOI:** 10.1021/acs.est.2c03559

**Published:** 2022-10-28

**Authors:** Lise M. Alkema, Caspar J. Van Lissa, Merel Kooi, Albert A. Koelmans

**Affiliations:** † Aquatic Ecology and Water Quality Management Group, Wageningen University, P.O. Box 47, 6700 DDWageningen, The Netherlands; ‡ Department Methodology and Statistics, Tilburg University, PO Box 90153, 5000 LETilburg, The Netherlands

**Keywords:** microplastic, macroplastic, plastic debris, Atlantic ocean, mixture models

## Abstract

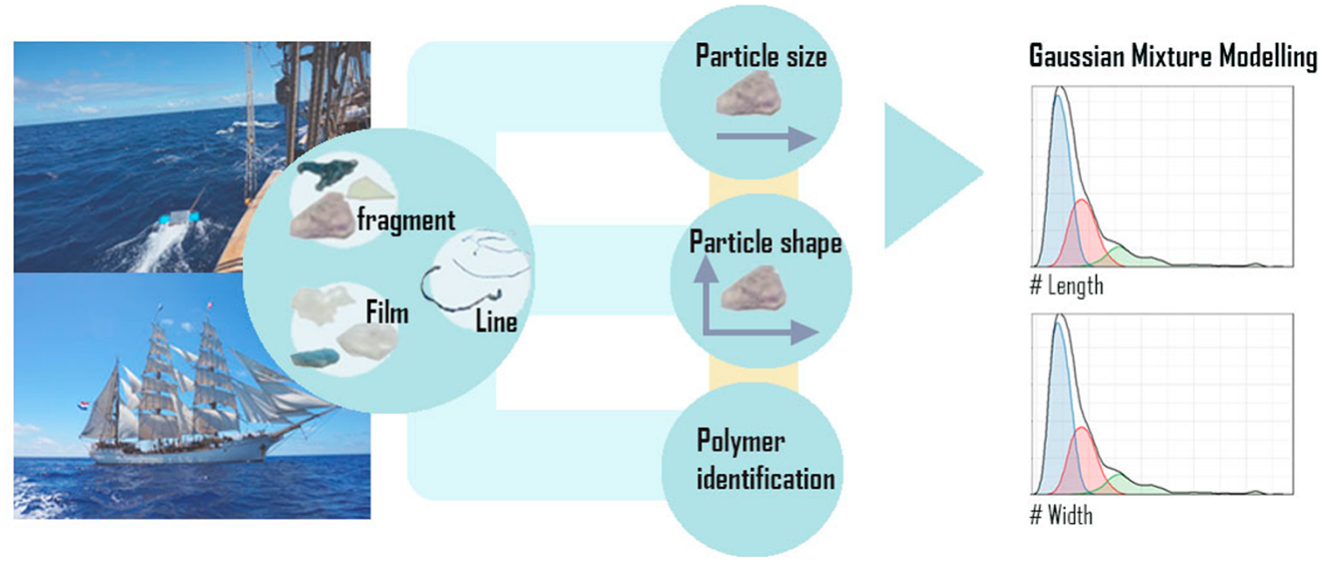

Current methods of characterizing plastic debris use arbitrary, predetermined categorizations and assume that the properties of particles are independent. Here we introduce Gaussian mixture models (GMM), a technique suitable for describing non-normal multivariate distributions, as a method to identify mutually exclusive subsets of floating macroplastic and microplastic particles (latent class analysis) based on statistically defensible categories. Length, width, height and polymer type of 6,942 particles and items from the Atlantic Ocean were measured using infrared spectroscopy and image analysis. GMM revealed six underlying normal distributions based on length and width; two within each of the lines, films, and fragments categories. These classes differed significantly in polymer types. The results further showed that smaller films and fragments had a higher correlation between length and width, indicating that they were about the same size in two dimensions. In contrast, larger films and fragments showed low correlations of height with length and width. This demonstrates that larger particles show greater variability in shape and thus plastic fragmentation is associated with particle rounding. These results offer important opportunities for refinement of risk assessment and for modeling the fragmentation and distribution of plastic in the ocean. They further illustrate that GMM is a useful method to map ocean plastics, with advantages over approaches that use arbitrary categorizations and assume size independence or normal distributions.

## Abstract 2

Latent class analysis of marine macro- and microplastic particles increases realism in describing classes of particles relative to previous predetermined and arbitrary classifications.

## Introduction

The persistence of plastic debris in the environment constitutes a threat to a multitude of life forms existing in the environment, including humans.^1,2^ This concern has given rise to numerous research initiatives that aim to map the amount, characteristics, and behavior of plastics that leak or have already leaked into the environment.^3^ Simultaneously, the potential risk associated with marine debris, including that of particles with a length smaller than 5 mm, also called “microplastics”, has sparked policy initiatives worldwide aiming to reduce plastic littering.^4−6^

In current research, data on plastics is often presented in the form of predetermined categories of a qualitative nature; descriptive categories of polymer type (e.g., polyethylene (PE), polypropylene (PP) or polystyrene (PS)), size (nanoplastic, microplastic, macroplastic), and shape (fragment, film, foam, pellet, line), some of which are rather arbitrary.^7,8^ These methods effectively describe and visualize characteristics of marine plastics; however, they are limited in providing quantifiable information on the heterogeneity within predetermined categories or size. Microplastic is a very diverse contaminant, but most studies fail to provide data on the material’s true multidimensionality and heterogeneity.^9^ Furthermore, methods used for data sampling and analysis in the relatively young field of plastic research are fragmented, making it hard to compare and contrast available data.^9−13^

A unified method of sampling and analysis is needed that provides a way to describe data while providing quantifiable information. Kooi and Koelmans (2019) have taken a first step in building new best practises.^10^ They propose to move away from the sole use of discrete classifications and to map the characteristics of plastics through continuous distributions.^10,12^ Besides capturing the heterogeneous multidimensional nature of plastics, parametrizations of these distributions allow for comparison between studies, irrespective of laboratory or sampling setups, and will be helpful in probabilistic risk modeling.^10,9,14−18^

However, to date such analysis only considered microplastics, which is a size category, and assumed size, shape, and density to be independent. A next step is to examine whether this assumption of independence holds, what distributions look like for a size range larger than that of microplastic and to validate Kooi and Koelmans’^10^ findings on a larger data set without using precalculated shape coefficients.

Gaussian finite mixture modeling (GMM) is a method used in other disciplines to build typologies, taxonomies, and classifications based on a set of (potentially correlated) measured characteristics.^19^ Over the past decade GMM has grown in use across several disciplines as a general modeling tool that accounts for heterogeneity in data, which only occasionally has been used in the natural sciences, e.g., for facies mapping from binary geological data,^20^ or to identify patterns of multiple co-occurring exposures to polycyclic aromatic hydrocarbons.^21^ It is useful for describing non-normal distributions of particle dimensions as a mixture of normal distributions. Simply put: the observed distribution, which is not normal, is modeled as a blend of several overlapping distributions, which *are* normal. The properties of these underlying normal distributions–means, standard deviations, and correlations–can be interpreted in the usual way, which offers a more nuanced understanding of the prevalence and properties of ocean plastics than descriptive statistics of a non-normal distribution.

The present study applies GMM to a data set of *n* = 6942 particles, ranging between 1 mm and 137.8 mm in length, obtained during a cruise on the North Atlantic Ocean. The aim of this paper is to map distributions of size, operationalized in terms of length and width, and shape, as close to reality as possible, looking only at our measured properties. We did not include color as a property, because color is less relevant to the mechanisms that determine the exposure and effects of plastic particles.^22^ We make use of our entire data set without discriminating between size classes. Importantly, our extensive data set and the use of GMM allows for correlation testing between size, shape, and polymer type. Correlations are assessed through the mixture modeling process by assigning each particle to a probability of belonging to each of those underlying normal distributions. This results in a latent classification of particles with similar length and width. The different classes can be compared with respect to auxiliary variables, for example, to determine whether polymer type differs across classes, or whether smaller particle length correlates with smaller particle width. With this analysis, a mixture model of normal distributions is constructed based on the latent classes detected, and its merits are discussed in the context of ocean microplastic fate modeling and risk assessment. Although not our primary aim, we also provide data on particle number and mass concentration and on polymer identity, to allow comparison with other data sets.

## Methods

In line with open science principles, all data and code for these analyses are available in a reproducible repository at https://github.com/cjvanlissa/lise_microplastics.

### Sampling Locations, Sampling Method, and Quality Control

**Figure 1:**
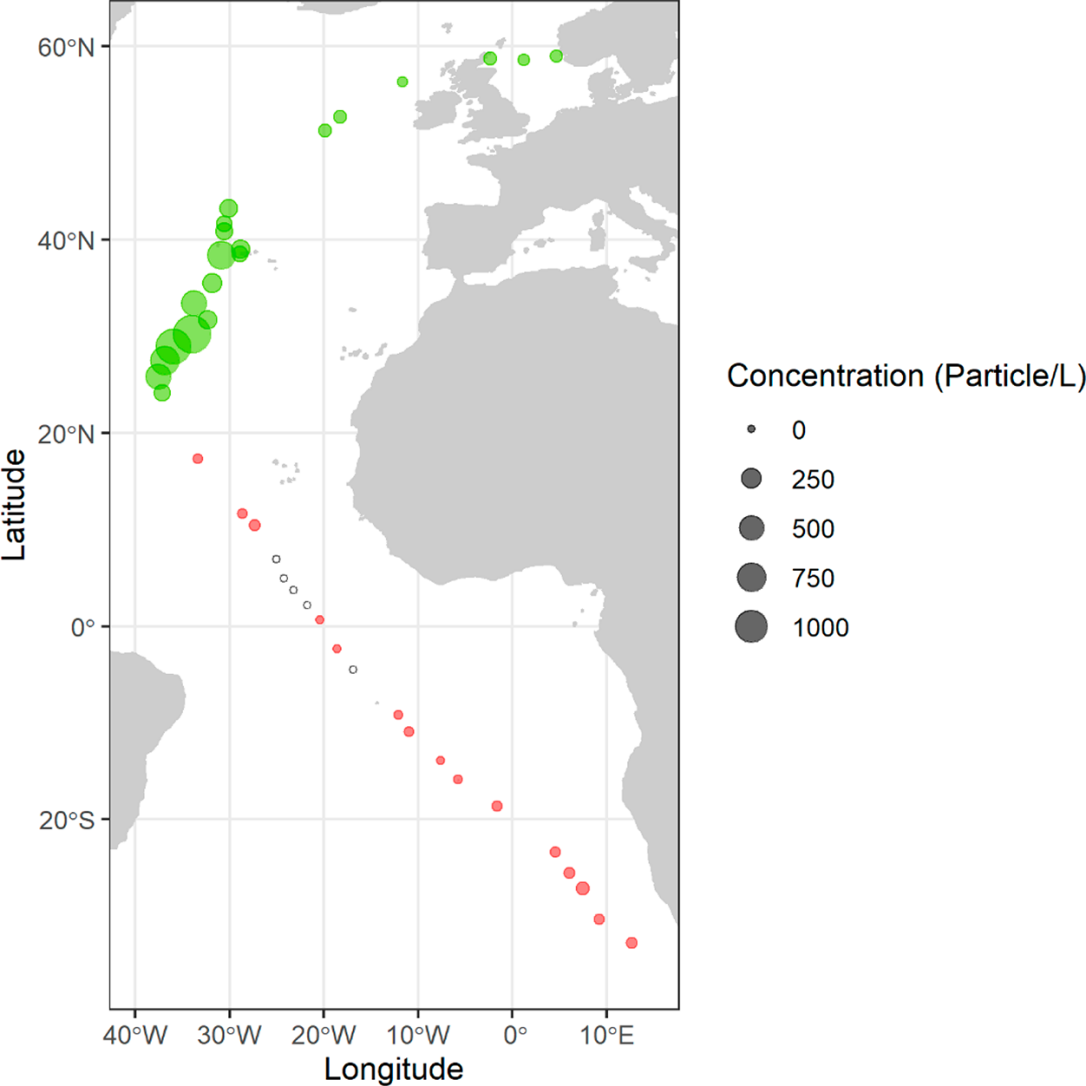
Plastic concentration per sample (particles/L) along our sampling route. The green color marks samples used for present GMM analysis. Red color marks samples not considered suitable for the GMM analysis, due to inconsistent sample conditions.

Plastic particles were collected during a cruise from 04/2018 to 06/2018 traversing the Atlantic Ocean from South Africa (Cape Town) to Norway (Stavanger). A total of 40 samples has been collected, of which 20 were in the South Atlantic and North Equatorial Current and 20 were in the remaining part of the North Atlantic (Figure 1). To this end, a 500 μm meshed Manta Trawl with an aperture of 15 × 85 cm was towed outside the wake of the ship for 1 h each day (when weather circumstances allowed). Wind speed, boat speed, and sea state were recorded during all samplings (Table S1). Maximum boat speed for reliable sampling was set at 5 knots an hour.

After each tow, the net was taken out of the water and rinsed with unfiltered seawater from the outside of the opening toward a detachable 500 μm cod-end, its content being emptied in a bucket and filtered through a 1 mm sieve to allow for visual inspection.^23,24^ The lower limit for particle detection was therefore 1 mm. For the targeted particles sizes >1 mm, background contamination can be considered negligible. From the sieve, particles were picked by hand aided by tweezers and magnifying glasses. Sometimes the net was obstructed by seaweed, jellyfish, or Syphonophores. In this case the full content of the manta net was placed in a bucket, after which we manually sorted through all seaweed, carefully placing each piece in seawater and checking thoroughly for remaining plastics before discarding. The sieve was always rinsed in a bucket of seawater, from which remaining particles were collected. After sieving and manual sorting, particles were dried and separated from organic matter by visual inspection, counted, and photographed. Hereafter, they were packed in either paper or aluminum foil and stored cool and dry to avoid biofouling and contamination. Particle number concentrations and mass concentrations were calculated. Sample volume (≈ 600 m^3^) was calculated as width of the trawl × sampled height underwater × sampled distance.^25^

For further analysis we selected 20 samples, the majority being from the center of the North Atlantic gyre and a minority from the North Atlantic Current and the North Sea (Figure 1). These had the highest relative abundancy, highest sample density, and highest reliability, controlling for external influences of sea state, wind state, and boat speed (Table S1). Because the sampling is done over a large area over a long period of time, with varying meteorological conditions (see Table S1), space-time differences are averaged out, and the pooled microplastic sample can be considered as a representative sample of the space-time variable population of microplastics on the ocean surface in the area.

When modeling the dimensionality of particles, it is important to consider inherent differences in the dimensions of different particle types. Therefore, particles were classified into several distinctive shape categories: “line”, “film” and “fragment”. Lines are defined as long, but with a relatively small and constant diameter. Films vary substantially along length and width, but are relatively flat (<0.5 mm). Fragments vary along all dimensions. Initially, we also used classifications of “foam” and “pellet”; however, these were reclassified under fragment, due to limited abundance (respectively 0.2% and 0.05% of all particles).

In most cases, the difference between organic matter and plastics was clear. Only film was less clear, as some seaweed produces a material with visual characteristics similar to plastics. To confirm our findings, all plastics of the 20 selected subsamples were identified using infrared spectroscopy (detailed below).^23^ For these samples, the visual method turned out to be rather accurate. Only 2% of the particles were identified as “other than plastic” or “NR” (not recognized by infrared spectroscopy).

### Laboratory Analysis

In the laboratory, cotton lab coats were worn and equipment and lab surfaces were wiped. Each sample was weighed, and an analysis was applied to identify polymer type and particle size (length, width, and, where possible, height) on an individual particle basis. (Table S2, Table S4).

#### Polymer Type

Given the high number of particles, efficiency was maximized by using a combination of spectroscopic techniques; near infrared spectroscopy (NIR) and Fourier transform infrared spectroscopy with attenuated total reflection (ATR-FTIR). Principles of plastic detection with NIR are described in numerous scientific papers (e.g., refs (26−28)). NIR is fast and accurate enough for the larger particles and items (>1.5 mm). ATR-FTIR is more laborious yet more accurate for smaller particles and film, which the NIR technique could not recognize. Black particles were excluded from IR analysis. NIR analysis was performed using sIRoPad GUT 04e (GUT Environmental Technologies) and IoSys sIRo (Dr. Timur Seidel e.K) NIR measurement systems for the absorption spectrum in the range of 12,500 to 4,000 cm^–1^. After NIR analysis, particles were categorized per sample, per polymer type, and photographed for image analysis (detailed below). Particles not recognized by NIR (*n* = 135, mostly film and particles <1.5 mm) were analyzed separately using a Bruker ATR-FTIR Compact spectrometer ALPHA II, for the absorption spectrum in the range of 700 to 4,000 cm^–1^. Given that ATR-FTIR is laborious, a random subsample of 50 particles was photographed and analyzed for samples exceeding more than 50 particles. Before each measurement, particle surfaces were cleaned with ethanol. Spectra were produced with six scans, and analyzed with the OMNIC Picta Software (Thermo Fisher Scientific), comparing spectra with multiple polymer libraries (Table S5).

#### Particle Length, Width, and Height

Data on length (maximum Feret diameter) and width (minimum Feret diameter) were obtained through image analysis using ImageJ.^29^ The superiority of Feret’s diameter as a proxy of size compared to bounding rectangle dimensions was demonstrated by testing 6 particles with differing positions 10 times using ImageJ’s manual measurement tool and comparing mean results per particle with the three parameters (Table S6). Different shape classes required different analysis. Lines are often twisted, therefore it is difficult to measure their length directly. To approximate length, we divided the measured area of lines by their width (mean = 0.65 mm, SD = 0.18 mm, range 1.1 mm, N = 47). The approximate height (mean = 0.009 mm, N = 10) of film was considered neglectable, and thus film is accurately described by the Feret length and width. For small fragments (all dimensions <5 mm), we were able to measure only Feret length and width, as height of particles cannot be obtained from 2D images. For larger particles, we additionally measured height using a ruler.

For the NIR analysis, particles were photographed per resulting class of polymer type, therefore data on length, width, and height could be linked to polymer type on a particle basis. For the ATR-FTIR analysis, an image was acquired first, after which the ATR-FTIR analysis was conducted following the exact order of particle position on the photo; hence, length and width could be linked to polymer type and organic matter could be extracted. Particles that were not included in the subsamples used for the ATR-FTIR were photographed and analyzed separately on length and width. For all particles, shape category (line, film, fragment) was determined again through visual inspection of the photographs used for image analysis (Figure S1). Here, film was distinguished from fragment by looking at the solidity of a particle: a solid particle was classified as fragment, whereas a nonsolid see-through particle was classified as film.

ImageJ particle analysis was automated using the ImageJ Macro Plugin created by Mutterer & Rasband.^30^ To enable automation, all photos were taken with a tripod with standardized conversion of 13 pixels per mm, determined through manual measurement in ImageJ of a photographed scale (ruler). In total, for all 6942 particles data on length, width, and shape category was obtained, of which 4841 particles could be linked to their respective identified polymer type.

Quality assurance and control (QA/QC) was evaluated and is provided as Supporting Information.

### Data Analysis (GMM)

Prior to GMM analyses, we removed outliers separately for particles in the three categories: lines, films, and fragments. For lines, outliers were identified as cases with an absolute standardized value >3 SD for length.^31^ For films and fragments, outliers were identified as having a Mahalanobis distance >13.82 for length and width.^32^

The GMM analyses represent the observed data on length and width as a mixture of several multivariate normal distributions. The number of multivariate normal distributions used corresponds to a predetermined number of unobserved latent classes, which ranged from one (corresponding to the assumption of multivariate normality) to a maximum of five in our analyses. The GMM simultaneously estimates the multivariate distributional properties (mean, standard deviation, and covariance) of each class, and the probability that each particle belongs to each of these classes. The results of this analysis thus consist of maximum likelihood estimates of the descriptive statistics of multivariate normal distributions for each class, as well as a posterior classification probability matrix. The latter can be used to determine class separability.

After the GMM analysis, several auxiliary analyses were performed. As observed height was only assessed for particles with one dimension exceeding 5 mm, it could not be included in the GMM. We did, however, explore relationships of the GMM classes with observed height as a distal outcome. Similarly, we explored relationships of length and width with polymer type.

#### Strategy of GMM Analysis

To account for the differences in particle dimensions, we used a stepwise plan of analysis: (a) analyze the three shape categories separately, using only the dimensions relevant for each category, (b) determine the best GMM model and number of classes with subsequent normal distributions for each dimensionality category, (c) create a joint GMM model, with number of classes and starting values based on step (b). We used starting values for the dichotomous indicator variables (dummy variables) to ensure lines and films are assigned to the correct classes. Because for *lines* there is one dimension (length), we conducted univariate mixture models, focusing on the dimension of length, estimating 1–5 classes with varying means and variances.^33^ Because for *film* there are two dimensions (length and width), we conducted bivariate mixture models of length and width, estimating 1–5 classes. We compared models with varying means and variances, to models that also included varying covariances. For the remaining particles (*fragments*), we conducted the same bivariate mixture models as for film, analyzing the length and width of particles. The correct number of classes for each category was determined based on a combination of five criteria. We preferred models with a lower Schwarz’s Bayesian information criterion (BIC) and a significant bootstrapped likelihood ratio test compared to models with a smaller number of classes, whose minimum posterior classification probability ideally did not fall below 0.90 (but note one exception), and whose smallest class contained at least 10% of the sample.^34^ Class separability was determined based on the entropy of the posterior class probability matrix, normalized from 0 to 1 (1 standing for perfectly separated classes), where useful outcomes for these criteria were complemented with visual inspection of the solutions. To combine the models for *lines*, *films*, and *fragments*, we estimated a six-class mixture model. Six classes were used because analysis of the three categories of particles revealed that a two-class solution best fit the data from each of those categories. To evaluate overall fit, these three models with each two classes were combined into one overall model. Based on the classes obtained for each dimensionality category, we used dummy variables to restrict potential class membership for the three categories to two classes each. Parameters involving width were not estimated for line classes. To account for nonindependence of observations due to the clustered sampling (i.e., observations originating from the same haul of the net), we used a sandwich estimator for the standard errors. GMM models were conducted and reported in R using the tidyLPA package,^35^ and models were estimated in Mplus.^36,37^

Subsequently, we assessed correlation between length, width, and polymer type by looking at the standardized covariances across the distinguished classes within the separate models. For this, we used the three-step method by Bakk & Vermunt, 2014 (for the general method, see Asparouhov & Muthén, 2014).^38,39^ Three-step methods first estimate the mixture model, and then use the posterior classification probabilities to obtain unbiased estimates of differences between classes in auxiliary variables.

## Results and Discussion

### Plastics in the North Atlantic and North Sea

**Figure 2:**
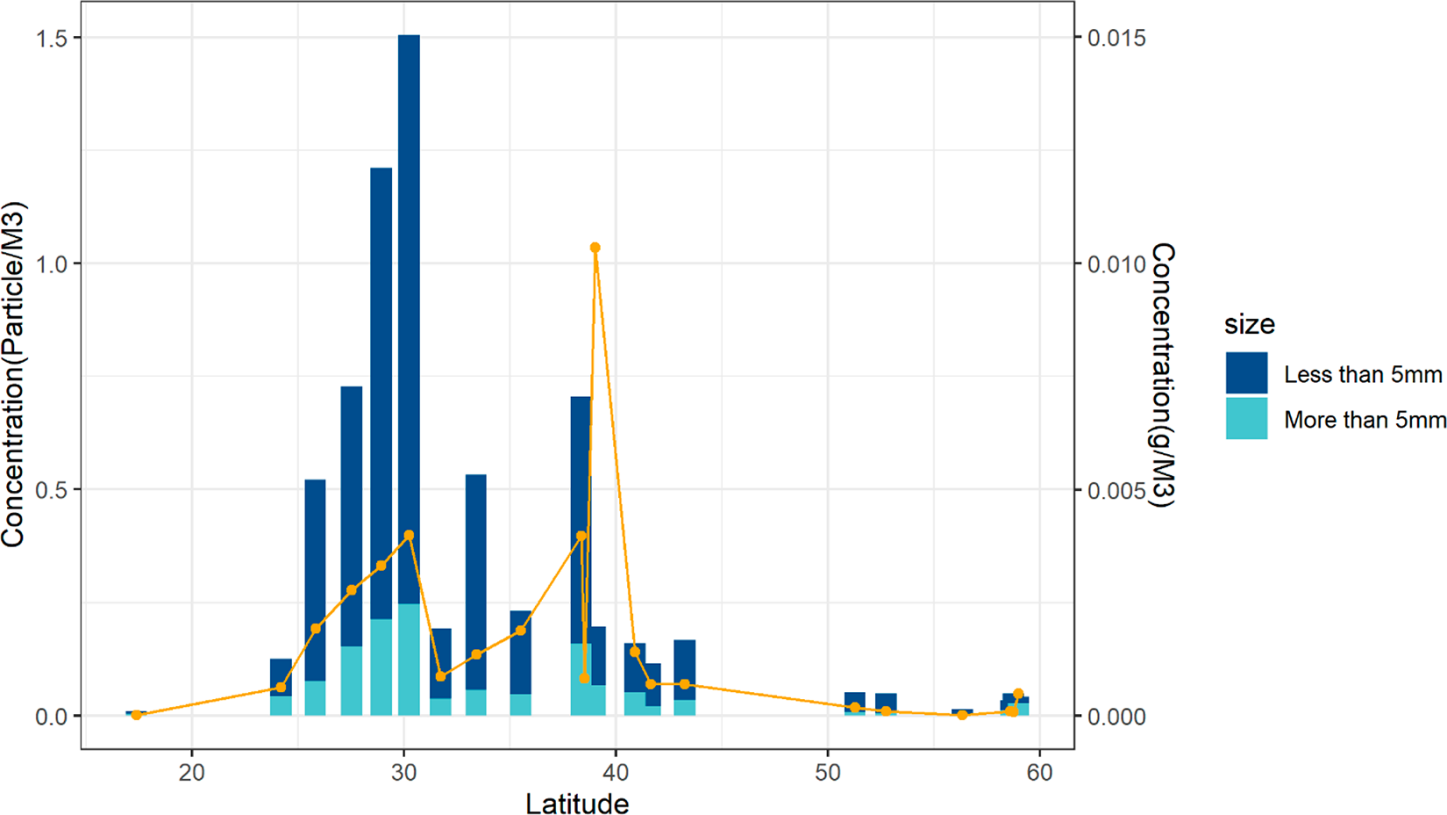
Particle concentration per sample (histogram) and mass concentration (yellow data points). Largest concentrations are found in the center of the North Atlantic (20°N–50°N), lower concentrations are found the North Atlantic Current (51°N–56°N), and in the North Sea (58°N). The outlier in mass concentration was caused by a bottle cap.

We observe high particle abundancy in a few samples, when crossing right through the middle of the center of the North Atlantic (close to the Azores) and lower concentrations in the North Atlantic Current and the North Sea (Figure 2). Highest concentrations were 1.5 particles/m^3^ and lowest concentrations were 0.009 particles/m^3^. An outlier in mass concentration was caused by a bottle cap (Figure 2). Differences in abundancies may be explained by the tendency of plastics to concentrate in the center of a gyre, making sample location of great influence to results.^40^ Besides, abundancies can be influenced by differences in favorable sea state conditions, which ranged from smooth to rough (Table S1).^41^ Indeed, rougher seas, more wind and high towing speed cause the net to destabilize, and buoyant particles can be pushed down and missed by the net.^42−44^ We experienced most favorable sea state conditions in the center of the North Atlantic, and less favorable conditions in the surrounding currents, which might influence the large differences in abundancies found (Table S1).

The vast majority of particles (81.9%) in our selected 20 samples fall into respectively the <5 mm “microplastic” size class category, and are classified for the largest part as fragments (80.5%) followed by film (12.3%) and line (7%) (Table S3). For polymer type, most particles were PE (polyethylene, 88%), followed by PP (polypropylene, 10.5%), and category “other” (0.7%) (Table S2). This last category contained particles identified as PS (polystyrene), PVC (polyvinyl chloride), PET (polyethylene terephthalate), PVA (polyvinyl acetate) and polyolefin (Table S4). Other studies report similar observations regarding the dominant prevalence of microplastics, fragments, PE, and PP among ocean plastics.^42,45−49^ However, other studies report higher amounts of foam and pellet than we found, and lower amounts of film and line. Similarly, studies researching the vertical distribution of ocean plastics or beach plastics indicate that a higher variety of particles might be prevalent in our ocean waters than only buoyant types (PE and PP) as these so-called high density types have a higher chance of sinking down over time, hereby being missed by surface trawling nets.^24,7,50^

Length shows a right skewed bimodal, and width shows a multimodal density distribution (Figure S2 and Figure S3). The bimodality and multimodality in our density distributions complicate correlation analysis, hence our decision to separate analyses for line, film, and fragment and to work with GMM. The majority of our data for length falls within 1–10 mm, with a maximum of 89.05 mm and a median of 2.59 mm (SD = 6.56). For width, we find a minimum of 0.2 mm, maximum of 16 mm, and median of 1.56 mm (SD = 1.28).

### Separate Gaussian Mixture Models for Each Dimensionality Category

#### Lines

**Figure 3:**
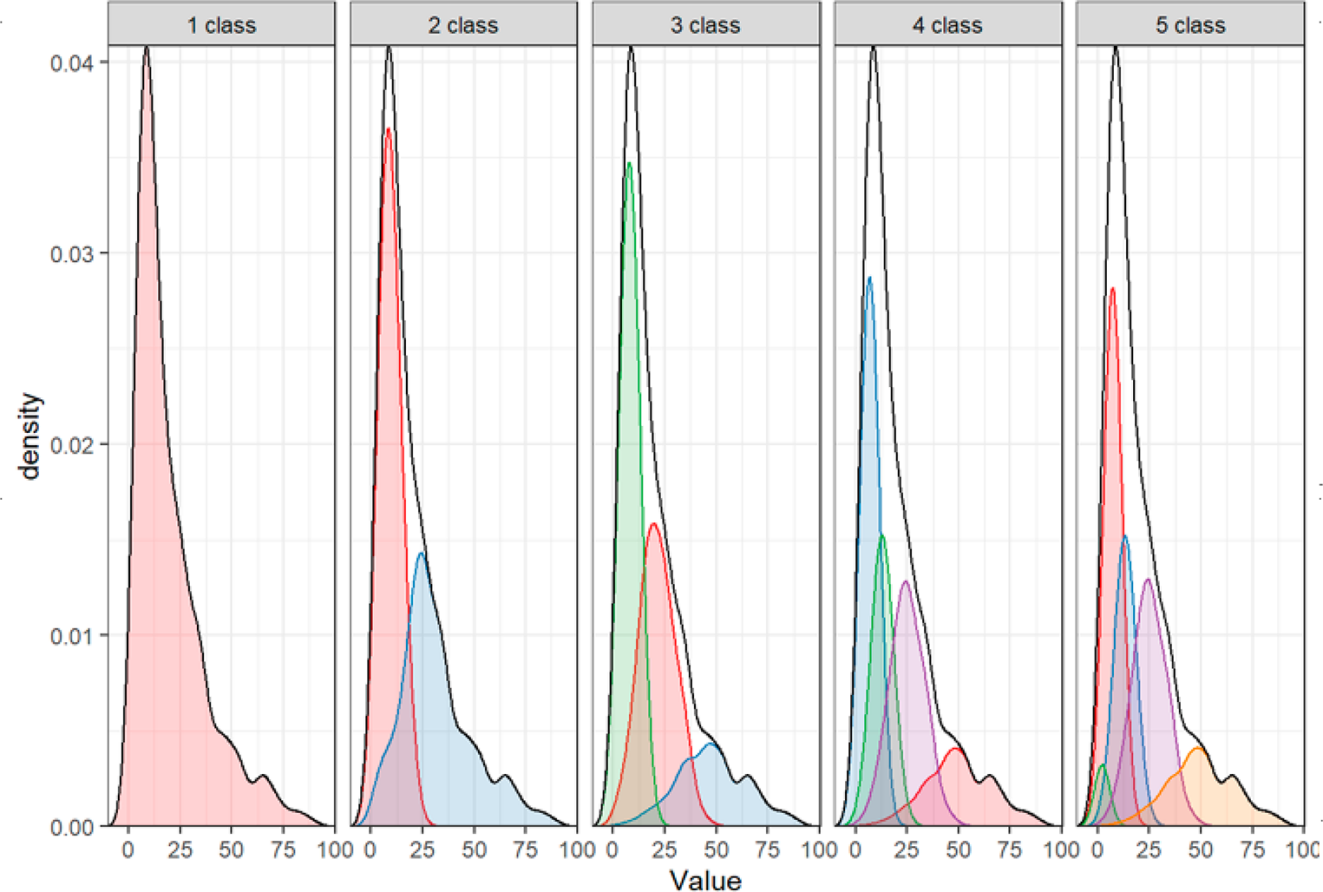
Comparison of mixture models for the length of lines (value; mm), estimating 1 to 5 classes with varying means and variances. Headers indicate the number of classes used in the mixture model. A 2-class solution is chosen, which shows one class of smaller lines, and one class of significantly larger lines. Large lines show a relatively high abundance.

For lines univariate mixture models were conducted, focusing on the dimension of length, estimating 1–5 classes with varying means and variances (Table S7). According to the BIC, a 3-class model fit best. However, the difference in BIC with the 2-class model was trivial, and the entropy of a 2-class solution was much higher. Most importantly, visual inspection of the solution (Figure 3) revealed a bimodal distribution. We thus chose a 2-class solution (Table S7).

#### Film

For film, we conducted bivariate mixture models of length and width, estimating 1–5 classes. Models with varying means and variances, were compared to models that also included varying covariances. BIC values hardly differed between the models with fixed and free covariances (Table S8). We thus prefer the simpler models with fixed covariances. Furthermore, BIC showed a substantial drop from the 1- to 2-class solution, followed by a smaller drop to the 3-class solution, after which the decrease stabilized. Entropy was higher for the 2-class than for the 3-class solution, indicating that the two classes were more clearly separable. Visual inspection of the solutions (Figure S4, Figure S5) was inconclusive. Per Occam’s razor, we thus retained the simpler 2-class solution (Table S8).

#### Fragments

**Figure 4:**
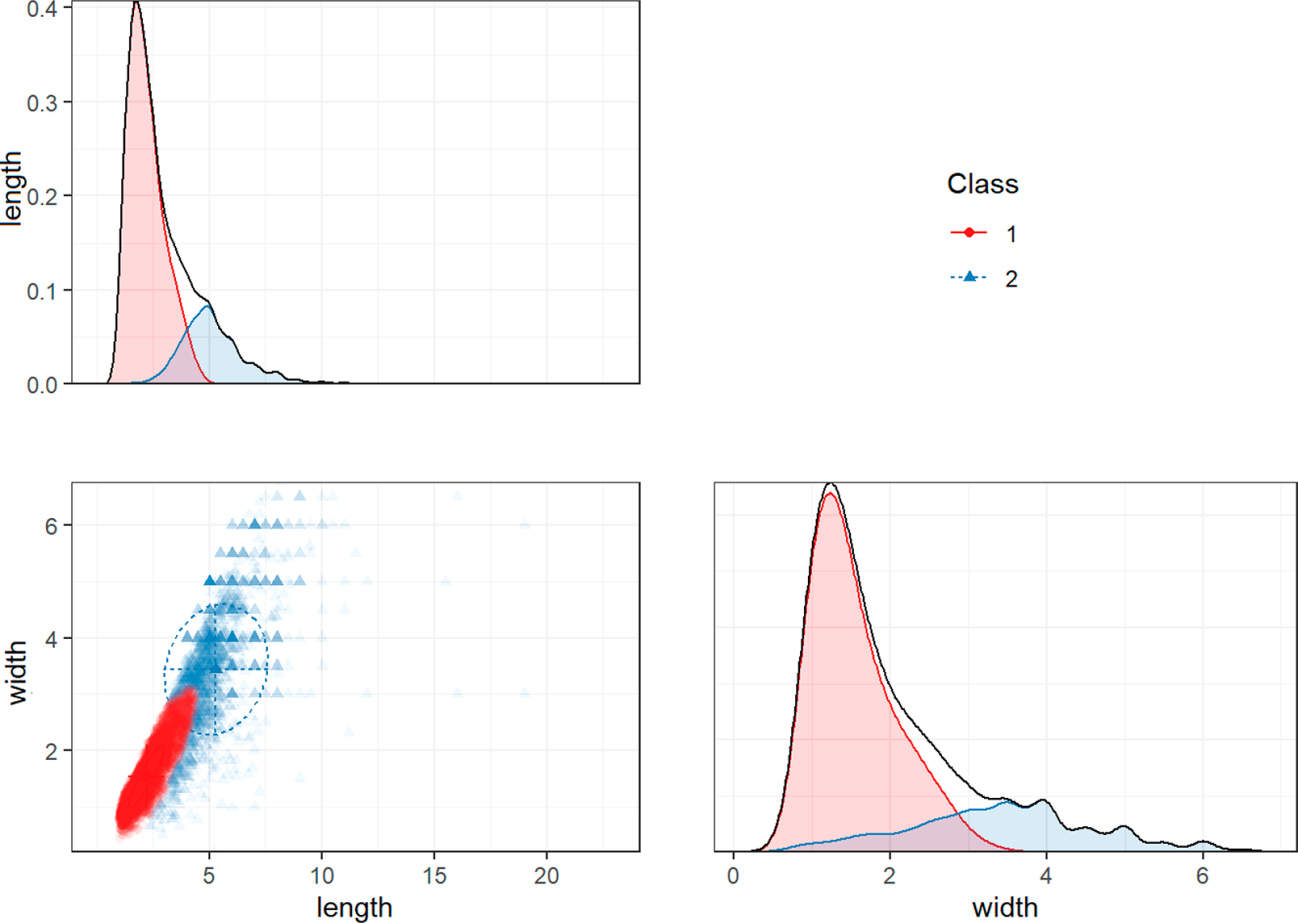
Mixture model of fragments with free means and variances, and fixed covariances. A 2-class solution shows a large class with a length that is relatively equal to width (strong correlation), and a smaller class with a length that is substantially different from width (weak correlation), indicating heterogeneity of shape for large fragments, and homogeneity of shape for small fragments. Length and width in mm.

For fragments, we conducted the same bivariate mixture models as for film, analyzing the length and width of particles. Similar to the models for film, BIC values hardly differed between the models with fixed, and free covariances (Table S9). We thus prefer the simpler models with fixed covariances. Furthermore, BIC showed a substantial drop from the 1- to 2-class solution, after which the decrease stabilized. Entropy was higher for the 3-class than for the 2-class solution; however, the lowest posterior classification probability is smaller than 0.80 and the smallest class only contains 5% of the data. This indicates that a three class solution is not preferable over a 2-cass solution. Visual inspection of the solutions (Figure S6) were inconclusive. Again, per Occam’s razor, we thus retained the simpler 2-class solution (Table S9, Figure 4).

### Classifying Floating Marine Debris through Gaussian Mixture Modeling

To combine the models for lines, films, and fragments, a six-class mixture model was estimated. Based on the above results, potential class membership for the three categories was restricted to two classes each. The resulting model discriminated well between classes (Entropy = 0.94, posterior classification probabilities [0.82, 0.99], Akaike Information Criterion (AIC) = 42760.41, BIC = 42945.24). The model parameters provide the features of the six mutually exclusive and exhaustive “latent” classes in which each of the individual particle samples from the North Atlantic can be classified (Table 1).

**Table 1:** Combined Model^a^

Parameter	Value (mm)	95% CI	Class
*M*_length_	9.32*	[8.20, 10.44]	Line 1 (*N* = 289)
*s*_length_^2^	19.87*	[10.08, 29.65]	
*M*_length_	32.36*	[29.66, 35.06]	Line 2 (*N* = 195)
*s*_length_^2^	312.35*	[247.17, 337.52]	
*r*_length_,_width_	0.86*	[0.84, 0.88]	Film 1 (*N* = 714)
*M*_length_	2.95*	[2.63, 3.26]	
*M*_width_	1.81*	[1.57, 2.05]	
*s*_length_^2^	1.70*	[1.25, 2.15]	
*s*_width_^2^	0.63*	[0.49, 0.76]	
*r*_length_,_width_	0.08*	[0.05, 0.12]	Film 2 (*N* = 139)
*M*_length_	9.29*	[8.50, 6.12]	
*M*_width_	5.60*	[5.08, 6.12]	
*s*_length_^2^	14.11*	[8.68, 19.55]	
*s*_width_^2^	7.89*	[5.25, 10.54]	
*r*_length_,_width_	0.91*	[0.88, 0.94]	Fragment 1 (*N* = 4338)
*M*_length_	2.21*	[1.93, 2.48]	
*M*_width_	1.55*	[1.36, 1.73]	
*s*_length_^2^	0.63*	[0.32, 0.95]	
*s*_width_^2^	0.31*	[0.15, 0.46]	
*r*_length_,_width_	0.15*	[0.07, 0.23]	Fragment 2 (*N* = 1267)
*M*_length_	5.29*	[4.69, 5.89]	
*M*_width_	3.44*	[3.15, 3.73]	
*s*_length_^2^	5.36*	[−2.56, 13.28]	
*s*_width_^2^	1.35*	[0.91, 1.80]	

a *M*: Mean; *s*^2^, variance; *r*, correlation. * = *p* < 0.001.

#### Length, Width, and Shape Category

The results indicated that, for all three shape categories, two classes could be distinguished, resulting in a total of six classes (Table 1, Figures 3, 4, and S4). For all shape categories one class with smaller means and variances was found, and one class with larger means and variances. For films and fragments, we were additionally able to estimate covariances in these classes. Although the covariances were fixed to be equal across the two classes, they are standardized differently because the variances are freely estimated across the two classes. Thus, for films and fragments, we observe different standardized covariances (i.e., correlation coefficients) between length and width in the two classes. These correlation coefficients inform us about the (two-dimensional) shape of particles. High correlations indicate that particles have a similar length and width, whereas low correlations reflect particles that are considerably longer than they are wide.

For films and fragments, these correlations between length and width were stronger in the classes with smaller particles than in the classes with larger particles. This implies that small films and fragments are approximately equally wide as they are long. By contrast, the correlation between length and width was near-zero for large films (*r* = 0.09), and moderate for large fragments (*r* = 0.43). These low correlations indicate that larger films and fragments show substantial heterogeneity of shape.

#### Observed Height

First, we examined the prevalence of observed height measurements for particles over 5 mm across the six particle classes, based on most likely class membership. We found that only 5 particles with observed height were not classified as *large fragments* (larger than 5 mm). Consequently, we analyzed observed height only for particles classified as *large fragments*. For these particles, we found that length was uncorrelated with height, *r* < 0.01, and width had a small correlation with height, *r* < 0.24. Along with the aforementioned lower correlation between length and width, this low correlation with height again reinforces the notion that larger particles display greater heterogeneity in shapes.

#### Polymer Type by Class

**Figure 5:**
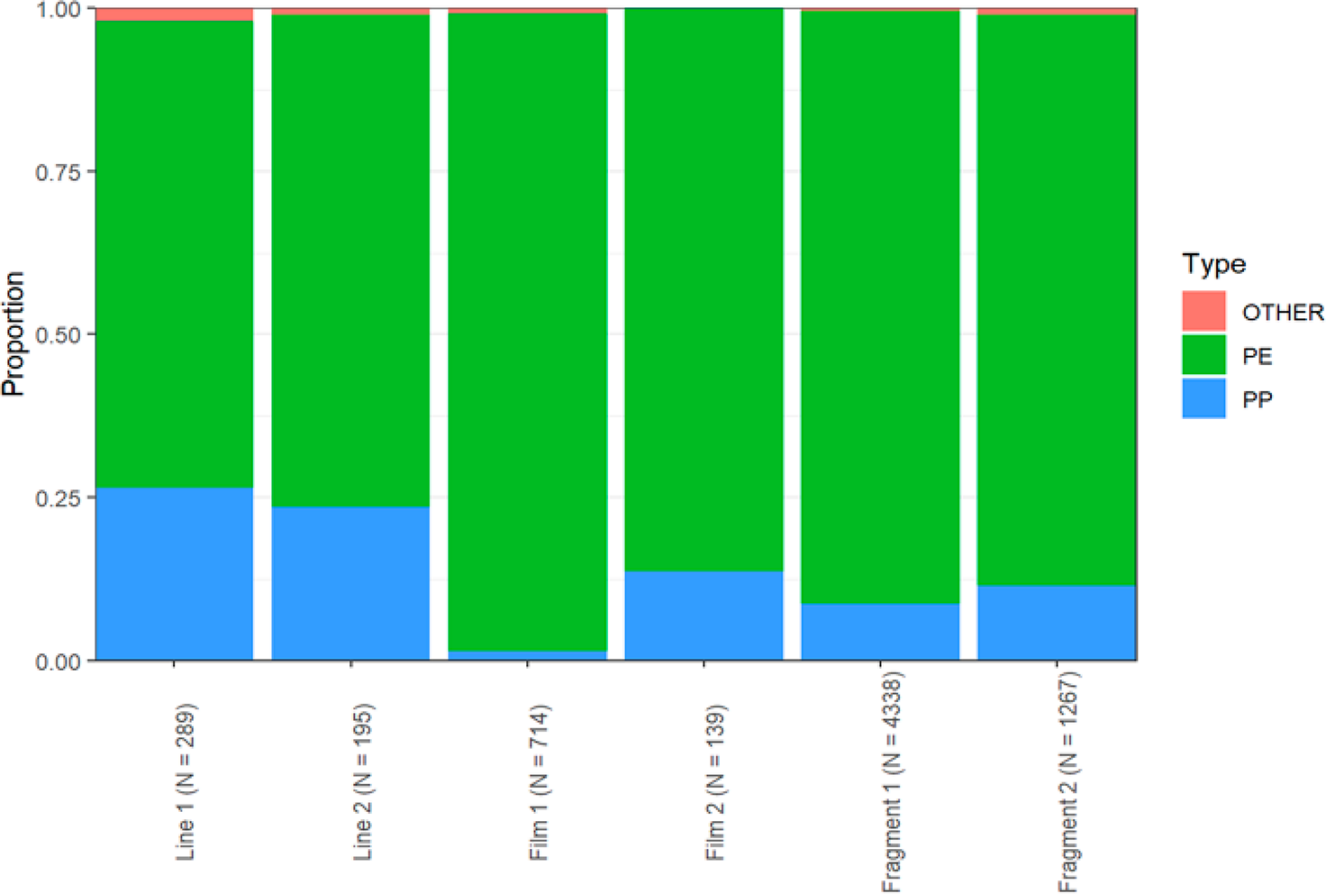
Proportion of polymer types by latent class. There are significant differences across all classes for all three polymer types, implying that the classes differed significantly from one another with respect to polymer composition.

Using the three step method by Bakk & Vermunt 2014, we found significant differences across classes for all three polymer types, with all χ2(5) > 95.42, *ps* < 0.001. The resulting distribution of polymer types by class is displayed in Figure 5. It appears that PE dominates polymer distribution in all of the classes, which is not surprising considering that PE constitutes 87% of all polymers within our data set.

### General Discussion and Prospect

#### Benefits of GMM

Our analyses suggest that the uni- and multivariate prevalence of ocean plastics is better described by a mixture of (multivariate) normal distributions than by a single (multivariate) normal distribution. Mathematical models of ocean plastic prevalence should take this deviation from (multivariate) normality into account. Simulating particle distributions from a mixture of Gaussian distributions, with parameters and proportions informed by GMM analyses, would be a computationally efficient and easy to understand way to account for this deviation from normality.

GMM has added value compared to basic correlation analysis. The advantage is that simple correlation analysis does not do justice to the fact that the correlation between dimensions can be different for particles of different size. If all data are lumped together you find average correlations; if you split using GMM you can detect that the correlation depends on the size of the particle. Our analysis indeed shows that size, shape, and polymer type of ocean plastics are not independent. For films and fragments, we found high correlations between length and width for classes of smaller particles, and low correlations for classes with large particles, indicating that shape of particles is significantly correlated with size. We demonstrate that larger particles show relatively more heterogeneity in shape, while smaller particles are more rounded, i.e., more cubical or spherical shaped. This is consistent with known breakdown mechanisms for rock particles like chipping and fragmentation.^51^ Chipping occurs at relatively low impact energies and includes shallow cracking; this process rounds off particles in a general way. Fragmentation occurs through catastrophic rupture due to fracture growth in the bulk, which requires high impact energies and produces angular shards.^51^ These findings illustrate how GMM can be used to assess the likelihood of degradation mechanisms. From a polymer chemistry and manufacturing point of view, it is further recommended to explore how GMM methods can link size, shape, and polymer-type correlations to properties of plastic products in general.

#### Limitations

However, some limitations can be noted. We initially conducted separate analyses for lines, which vary primarily along the length dimension, films, which vary primarily along the length and width dimensions, and fragments, which vary in all three dimensions. Films, by definition, have limited variability in height. Thus, their shape is adequately described by the correlation between length and width. Fragments vary in height, but we had only limited measurements of height. We therefore cannot make definitive claims about the three-dimensional shape of fragments. Moreover, our height measurements were biased, because data was primarily missing for smaller particles. Extrapolating from the very high correlation between length and width (*r* < 93) observed in small fragments, we speculate that, for small fragments, the correlation of these dimensions with height will also be large. Future research should investigate whether small fragments are indeed approximately coextensive in three dimensions. For large fragments, correlations of height with length (*r* < 01) and width (*r* = 0.24) were small, which indicates that these fragments show substantial variability in three-dimensional shape. More generally, the present findings are constrained by the sampling and measurement methods used. Our results might overestimate the concentration of (smaller) particles, as particles are brittle and break easily during laboratory analysis. Furthermore, future work may improve the shape and polymer characterization approaches and should extend the size range to smaller as well as larger scale. Smaller fractions than the 1 mm studied here are also very interesting for the microplastics community, as the bioaccessibility and effects of microplastics can increase when particles are smaller.^9,12,16,18^ The method introduced here could therefore be applied to smaller microplastics, and nanoplastics, but also macroplastics for which we have already been able to cover sizes up to 9 cm. This makes it possible to see to what extent the current conclusions apply to the size ranges that we have not explored here, which will lead to an increasingly better understanding of the nature of environmental plastics across size scales.

#### Advancing the Risk Assessment of Microplastic Particles

Risk assessment requires that the description of the particle properties remains as close as possible to how the properties of microplastic mixtures occur in nature. Our results provide mutually exclusive classes of particles based on a statistical analysis of empirical data. The latter implies that, after the initial categorization of particles into line, film, and fragment,—a process that is necessary considering the inherent differences in shape dimensions between the categories—the classification is retrospective, remains completely true to the material as it occurs in nature, and can be considered unbiased. These actual classes contrast with the current public and academic focus on predetermined categories such as those referred to as for instance *microplastics*, *mesoplastic*, and/or *macroplastic*.^7^ The term “microplastic” puts a rather subjective, random, and exclusive limit on particles with a length smaller than 5 mm. Our analysis reveals that statistically meaningful classes of particles range beyond the arbitrary 5 mm cutoff. These particles are likely to have similar types of harmful effects on marine organisms as “microplastics”, as long as they are ingestible.^9^ Recent work has suggested that food dilution is a relevant effect mechanism for plastic particles and this mechanism is not likely to be less relevant for a 6 mm particle compared to a 5 or 4 mm particle.^9,15,22^ Retrospective risk assessments can be based on factual distributions that are categorized afterward. We focused on micro- and mesoplastics as they have the highest risk profile with regard to biota uptake. However, the same GMM methods could be applied to wider size ranges. This would affect the models as such: other and more latent classes would be obtained, with different parameters. Follow-up studies can build on this, e.g., by applying the presented method to macroplastic particles and items larger in size.

The here outlined approach can be compared with that of Kooi and Koelmans,^10^ who provided a hybrid of retrospective and prospective assessments of classes of particles. They *retrospectively* analyzed particle size distributions based on empirical data from literature which however were not detailed enough to allow for subclassification. For shape and polymer identity, however, a *prospective* approach was used. A priori knowledge on shape categories and polymer identities of microplastic particles was combined with known average relative abundance data in order to create particle classes, which were combined to construct overall distributions.^10^ In a way this is the reversed procedure of our present analysis, where latent classes of particles are calculated back from known distributions of characteristics. With the earlier approach, however,^10^ correlations among characteristics could not be taken into account, whereas the distributions remained within the microplastic size range of 1 μm to 5 mm. The difference between the two procedures may also relate to the type of risk assessments in which they could be applied. Retrospective risk assessment for plastic debris would consider as much as possible the full realism of particles, exposed organisms, and site characteristics for which the assessment has to apply given the problem definition of the assessment.^15^ GMM models could play a relevant role in such assessments by defining what are the relevant groups of particles that should be taken into account. Prospective risk assessments would not necessarily be site specific and could be more generic, which implies that a priori definition of particle groups could apply in many cases.

Application of Gaussian mixture models in probabilistic retrospective risk assessments would imply Monte Carlo simulations where the multidimensionality of microplastics is captured by repeated random sampling of values for microplastic characteristics (e.g., length, width, size, polymer identity) from the mixture of (multivariate) normal distributions. Such an overall distribution would take the shape
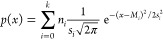
1where *p*(*x*) is the probability density function for particle characteristic *x* (for instance particle length, width, or length/width), *k* is the number of classes taken into consideration (e.g., *k* = 6, see Table 1), *n*_*i*_ is the number fraction of particles in class *i*, *s*_*i*_ is the standard deviation for class *i*, and *M_i_* the mean of the distribution for class *i*. The number fraction of particles in class *i* is calculated as *n*_*i*_ = *N*_*i*_/*N*_total_, with *N*_*i*_ being the number of particles in class *i*, and *N*_total_ being the total number of particles in all classes taken into consideration. Parameter values for *k*, *N*_*i*_, *N*_total_, *M*_*i*_, and *s*_*i*_ are those as provided in Table 1. Since the errors in these parameters are known (Table 1), uncertainty in the parameters can also be taken into account probabilistically. As recently demonstrated, having mathematical equations for ecologically realistic mixtures of microplastics (i.e., eq 1) offers great opportunities to quantify and align the ecologically relevant metrics (ERMs) used in microplastic risk characterization.^9,11,12,14−18^

## Supporting Information Available

Sampling conditions and overall count; abundancies and frequencies of categories per polymer type; abundancies and frequencies of shape categories; findings for polymer type category “other”; polymer type libraries; manual length and width measurements versus Ferrets diameter and bounding rectangle measurements; line classification; film classification; fragment classification; comparison of Ferrets diameter with bounding rectangle diameter; density distribution for particle length for film, fragment and line; density distribution for particle width; mixture model of film (2 class solution); mixture model of film (3 class solution); mixture model of fragments (3 class solution) (PDF)

## References

[ref1] Science Advice for Policy by European Academies. A Scientific Perspective on Microplastics in Nature and Society; SAPEA: Berlin, 2019.

[ref2] Microplastics in drinking-water; World Health Organization: Geneva, 2019. License: CC-BY-NC-SA 3.0 IGO.

[ref3] Law K. L. Plastics in the Marine Environment. Annu. Rev. Mar. Sci. 2017, 9, 205–209. 10.1146/annurev-marine-010816-060409. 27620829

[ref4] Xanthos D.; Walker T. R. International policies to reduce plastic marine pollution from single-use plastics (plastic bags and microbeads): A review. Mar. Pollut. Bull. 2017, 118 (1–2), 17–26. 10.1016/j.marpolbul.2017.02.048. 28238328

[ref5] Vince J.; Hardesty B. D. Plastic pollution challenges in marine and coastal environments. From local to global governance. Restor. Ecol. 2017, 25, 123–12. 10.1111/rec.12388.

[ref6] Syberg K.; Nielsen M. B.; Westergaard Clausen L. P.; van Calster G.; van Wezel A.; Rochman C.; Koelmans A. A.; Cronin R.; Pahl S.; Hansen S. F. Regulation of plastic from a circular economy perspective. Curr. Opin. Green Sustain. Chem. 2021, 29, 100462 10.1016/j.cogsc.2021.100462.

[ref7] Hartmann N. B.; Hüffer T.; Thompson R. C.; Hassellöv M.; Verschoor A.; Daugaard A. E.; Rist S.; Karlsson T.; Brennholt N.; Cole M.; Herrling M. P.; Hess M. C.; Ivleva N. P.; Lusher A. L.; Wagner M. Are We Speaking the Same Language? Recommendations for a Definition and Categorization Framework for Plastic Debris. Environ. Sci. Technol. 2019, 53, 1039–1047. 10.1021/acs.est.8b05297. 30608663

[ref8] Rochman C. M.; Brookson C.; Bikker J.; Djuric N.; Earn A.; Bucci K.; Athey S.; Huntington A.; McIlwraith H.; Munno K.; De Frond H.; Kolomijeca A.; Erdle L.; Grbic J.; Bayoumi M.; Borrelle S. B.; Wu T.; Santoro S.; Werbowski L. M.; Zhu X.; Giles R. K.; Hamilton B. M.; Thaysen C.; Kaura A.; Klasios N.; Ead L.; Kim J.; Sherlock C.; Ho A.; Hung C. Rethinking microplastics as a diverse contaminant suite. Environ. Toxicol. Chem. 2019, 38, 703–711. 10.1002/etc.4371. 30909321

[ref9] Koelmans A. A.; Redondo-Hasselerharm P. E; Mohamed Nor N. H.; Kooi M. Solving the non-alignment of methods and approaches used in microplastic research in order to consistently characterize risk. Environ. Sci. Technol. 2020, 54 (19), 12307–12315. 10.1021/acs.est.0c02982. 32885967 PMC7547870

[ref10] Kooi M.; Koelmans A. A. Simplifying microplastic via continuous probability distributions for size, shape and density. Environ. Sci. Technol. Letters 2019, 6, 551–557. 10.1021/acs.estlett.9b00379.

[ref11] Koelmans A. A.; Besseling E.; Foekema E.; Kooi M.; Mintenig S.; Ossendorp B. C.; Redondo-Hasselerharm P. E.; Verschoor A.; van Wezel A. P.; Scheffer M. Risks of Plastic Debris: Unravelling fact, opinion, perception and belief. Environ. Sci. Technol. 2017, 51, 11513–11519. 10.1021/acs.est.7b02219. 28971682 PMC5677762

[ref12] Koelmans A. A.; Redondo-Hasselerharm P. E.; Mohamed Nor N. H.; de Ruijter V. N.; Mintenig S. M.; Kooi M. Risk Assessment of Microplastic Particles. Nat. Rev. Mater. 2022, 7, 138–152. 10.1038/s41578-021-00411-y.

[ref13] Cowger W.; Booth A. M.; Hamilton B. M.; Thaysen C.; Primpke S.; Munno K.; Lusher A. L.; Dehaut A.; Vaz V. P.; Liboiron M.; Devriese L. I.; Hermabessiere L.; Rochman C.; Athey S. N.; Lynch J. M.; De Frond H.; Gray A.; Jones O. A. H.; Brander S.; Steele C.; Moore S.; Sanchez A.; Nel H. Reporting Guidelines to Increase the Reproducibility and Comparability of Research on Microplastics. Appl. Spectrosc. 2020, 74, 1066–1077. 10.1177/0003702820930292. 32394727 PMC8216484

[ref14] Mohamed Nor N. H.; Kooi M.; Diepens N. J.; Koelmans A. A. Lifetime accumulation of nano- and microplastic in children and adults. Environ. Sci. Technol. 2021, 55, 5084–5096. 10.1021/acs.est.0c07384. 33724830 PMC8154366

[ref15] Kooi M.; Primpke S.; Mintenig S. M.; Lorenz C.; Gerdts G.; Koelmans A. A. Characterizing microplastics across environmental compartments. Water Res. 2021, 202, 117429 10.1016/j.watres.2021.117429. 34304075

[ref16] Mehinto A. C.; Coffin S.; Koelmans A. A.; Brander S. M.; Wagner M.; Thornton Hampton L. M.; Burton G. A.; Miller E.; Gouin T.; Weisberg S. B.; Rochman C. M. Risk-Based Management Framework for Microplastics in Aquatic Ecosystems. Microplast. Nanoplast. 2022, 17 10.1186/s43591-022-00033-3.

[ref17] Coffin S.; Weisberg S. B.; Rochman C. M.; Kooi M.; Koelmans A. A. Risk Characterization of Microplastics in San Francisco Bay, California. Micropl.&Nanopl. 2022, 2, 19 10.1186/s43591-022-00037-z.

[ref18] Redondo-Hasselerharm P. E.; Rico A.; Koelmans A. A. Risk assessment of microplastics in freshwater benthic ecosystems guided by strict quality criteria and data alignment methods. J. Hazard. Mater. 2023, 441, 129814 10.1016/j.jhazmat.2022.129814. 36075174

[ref19] Scotto Rosato N.; Baer J. C. Latent Class Analysis: A Method for Capturing Heterogeneity. Social Work Research 2012, 36, 61–69. 10.1093/swr/svs006.

[ref20] Doveton J. H.; Chang T. Latent Facies Mapping from Binary Geological Data. J. Geol. 1991, 99, 299 10.1086/629490.

[ref21] Hendryx M.; Luo J. Latent class analysis of the association between polycyclic aromatic hydrocarbon exposures and body mass index. Environ. Internat. 2018, 121, 227–231. 10.1016/j.envint.2018.09.016. 30218960

[ref22] de Ruijter V. N.; Redondo-Hasselerharm P. E.; Gouin T.; Koelmans A. A. 2020. Quality criteria for microplastic effect studies in the context of risk assessment: A critical review. Environ. Sci. Technol. 2020, 54 (19), 11692–11705. 10.1021/acs.est.0c03057. 32856914 PMC7547869

[ref23] Lenz R.; Enders K.; Stedmon C. A.; Mackenzie D. M. A.; Nielsen T. G. A critical assessment of visual identification of marine microplastic using Raman spectroscopy for analysis improvement. Mar. Pollut. Bull. 2015, 100, 82–91. 10.1016/j.marpolbul.2015.09.026. 26455785

[ref24] Rocha-Santos T.; Duarte A. C. A critical overview of the analytical approaches to the occurrence, the fate and the behaviour of microplastics in the environment. TrAC Trends Analyt. Chem. 2015, 65, 47–53. 10.1016/j.trac.2014.10.011.

[ref25] Pasquier G.; Doyen P.; Kazour M.; Dehaut A.; Diop M.; Duflos G.; Amara R. Manta Net: The Golden Method for Sampling Surface Water Microplastics in Aquatic Environments. Front. Environ. Sci. 2022, 10, 811112 10.3389/fenvs.2022.811112.

[ref26] Masoumi H.; Safavi S. M.; Khani Z. Identification and Classification of Plastic Resins using Near Infrared Reflectance Spectroscopy. Int. J. Mech. Ind. Eng. 2012, 6, 877–884.

[ref27] Zhu S.; Chen H.; Wang M.; Guo X.; Lei Y.; Jin G. Plastic solid waste identification system based on near infrared spectroscopy in combination with support vector machine. Adv. Ind. Eng. Polym. Res. 2019, 2, 77–81. 10.1016/j.aiepr.2019.04.001.

[ref28] Xia J.; Huang Y.; Li Q.; Xiong Y.; Min S. Convolutional neural network with near-infrared spectroscopy for plastic discrimination. Environ. Chem. Lett. 2021, 19, 3547–3555. 10.1007/s10311-021-01240-9.

[ref29] Schneider C. A.; Rasband W. S.; Eliceiri K. W. NIH Image to ImageJ: 25 years of image analysis. Nat. Methods 2012, 9, 671–675. PMID 22930834 10.1038/nmeth.2089. 22930834 PMC5554542

[ref30] ImageJ Macro Reference Guide. https://imagej.nih.gov/ij/docs/macro_reference_guide.pdf (accessed 01–08–2019).

[ref31] Tabachnick B. G.; Fidell L. S.; Ullman J. B. Using multivariate statistics, 7th ed.; Pearson: New York NY, 2019; 815 pages.

[ref32] Geun Kim M. Multivariate outliers and decompositions of Mahalanobis distance. Commun. Stat. Theory Methods. 2000, 29, 1511–1526. 10.1080/03610920008832559.

[ref33] Nylund K. L.; Asparouhov T.; Muthén B. O. Deciding on the number of classes in latent class analysis and growth mixture modeling: A Monte Carlo simulation study. Struct. Equ. Modeling 2007, 14, 535–569. 10.1080/10705510701575396.

[ref34] Van de Schoot R. Latent trajectory studies: The basics, how to interpret the results, and what to report. Eur. J. Psychotraumatol. 2015, 6, 27514 10.3402/ejpt.v6.27514. 25735413 PMC4348410

[ref35] R Core Team. R: A Language and Environment for Statistical Computing; R Foundation for Statistical Computing, 2020. https://www.R-project.org/ (accessed 01-07-2019).

[ref36] Rosenberg J.; Beymer P.; Anderson D.; Van Lissa C. J.; Schmidt J. tidyLPA: An R Package to Easily Carry Out Latent Profile Analysis (LPA) Using Open-Source or Commercial Software. J. Open Source Softw. 2018, 3, 978 10.21105/joss.00978.

[ref37] Muthén L. K.; Muthén B. O. Mplus User’s Guide: Vol., 7; Muthén & Muthén: Los Angeles, CA, 1998; 950 p.

[ref38] Bakk Z.; Vermunt J. K. Robustness of stepwise latent class modeling with continuous distal outcomes. Struct. Equ. Modeling 2016, 23, 20–31. 10.1080/10705511.2014.955104.

[ref39] Asparouhov T.; Muthén B. Auxiliary Variables in Mixture Modeling: Three-Step Approaches Using Mplus. Struct. Equ. Modeling 2014, 21, 329–341. 10.1080/10705511.2014.915181.

[ref40] Cózar A.; Echevarría F.; González-Gordillo J. I.; Irigoien X.; Úbeda B.; Hernández-León S.; Palma Á. T.; Navarro S.; García-de-Lomas J.; Ruiz A.; Fernández-de-Puelles M. L.; Duarte C. M. Plastic debris in the open ocean. Proc. Natl. Acad. Sci. U.S.A. 2014, 111, 10239–10244. 10.1073/pnas.1314705111. 24982135 PMC4104848

[ref41] Van Sebille E.; Wilcox C.; Lebreton L.; Maximenko N.; Hardesty B. D.; van Franeker J. A.; Eriksen M.; Siegel D.; Galgani F.; Law K. L. A global inventory of small floating plastic debris. Environ. Res. Lett. 2015, 10, 124006 10.1088/1748-9326/10/12/124006.

[ref42] Kukulka T.; Proskurowski G.; Morét-Ferguson S.; Meyer D. W.; Law K. L. The effect of wind mixing on the vertical distribution of buoyant plastic debris. Geophys. Res. Lett. 2012, 39, L07601 10.1029/2012GL051116.

[ref43] Eriksen M.; Lebreton L. C.; Carson H. S.; Thiel M.; Moore C. J.; Borerro J. C.; Galgani F.; Ryan P. G.; Reisser J. Plastic Pollution in the World’s Oceans: More than 5 Trillion Plastic Pieces Weighing over 250,000 Tons Afloat at Sea. PLoS One 2014, 9, e111913 10.1371/journal.pone.0111913. 25494041 PMC4262196

[ref44] Reisser J.; Slat B.; Noble K.; du Plessis K.; Epp M.; Proietti M.; de Sonneville J.; Becker T.; Pattiaratchi C. The vertical distribution of buoyant plastics at sea: an observational study in the North Atlantic Gyre. Biogeosciences 2015, 12, 1249–1256. 10.5194/bg-12-1249-2015.

[ref45] Morét-Ferguson S.; Law K. L.; Proskurowski G.; Murphy E. K.; Peacock E. E.; Reddy C. M. The size, mass and composition of plastic debris in the western North Atlantic Ocean. Mar. Pollut. Bull. 2010, 60, 1873–1878. 10.1016/j.marpolbul.2010.07.020. 20709339

[ref46] Galloway T. S.; Cole M.; Lewis C. Interactions of microplastic debris throughout the marine ecosystem. Nature Ecol. Evol. 2017, 1, 0116 10.1038/s41559-017-0116. 28812686

[ref47] McDermid K. J.; McMullen T. L. Quantitative analysis of small-plastic debris on beaches in the Hawaiian archipelago. Mar. Pollut. Bull. 2004, 48, 790–794. 10.1016/j.marpolbul.2003.10.017. 15041436

[ref48] Yokota K.; Waterfield H.; Hastings C.; Davidson E.; Kwietniewski E.; Wells B. Finding the missing piece of the aquatic plastic pollution puzzle: Interaction between primary producers and microplastics. Limnol. & Oceanogr. 2017, 2, 91–104. 10.1002/lol2.10040.

[ref49] Hidalgo-Ruz V.; Gutow L.; Thompson R. C.; Thiel M. Microplastics in the Marine Environment: A Review of the Methods Used for Identification and Quantification. Environ. Sci. Technol. 2012, 46, 3060–3075. 10.1021/es2031505. 22321064

[ref50] Ivar do Sul J.; Costa M. F.; Fillmann G. Microplastics in the pelagic environment around oceanic islands of the Western Tropical Atlantic Ocean. Water, Air, & Soil Poll. 2014, 225, 2004 10.1007/s11270-014-2004-z.

[ref51] Bodek S.; Jerolmack D. J. Breaking down chipping and fragmentation in sediment transport: the control of material strength. Earth Surf. Dynam. 2021, 9, 1531–1543. 10.5194/esurf-9-1531-2021.

