# Supplementary material for: Maximizing Realism: Mapping Plastic Particles at the Ocean Surface Using Mixtures of Normal Distributions
Source: Environ Sci Technol. 2022 Oct 28;56(22):15552–62. doi: 10.1021/acs.est.2c03559 (PMC9670840; doi:10.1021/acs.est.2c03559)
Supplement: Supplementary file 1 — es2c03559_si_001.pdf [file es2c03559_si_001.pdf]

Supporting Information

Maximizing realism: mapping marine plastics using mixtures of normal distributions

Lise Alkema[#,*], Caspar J. van Lissa[$], Merel Kooi[#] & Albert A. Koelmans[#]

[#] Aquatic Ecology and Water Quality Management Group, Wageningen University, P.O. Box 47, 6700 DD, Wageningen.

[$] Department of Methodology and Statistics, Utrecht University, the Netherlands

[*] Corresponding author: l.m.alkema@tue.nl

15 pages

9 Tables

6 Figures

Quality Assurance and control (QA/QC)

QA/QC Screening according to

Koelmans, A.A., Mohamed Nor, N.H., Hermsen, E., Kooi, M., Mintenig, S.M., De France, J. 2019. Microplastics in Freshwaters and Drinking Water: Critical Review and Assessment of Data Quality. *Water Research,* 155, 410-422. https://doi.org/10.1016/j.watres.2019.02.054

	Screening criterion	Motivation for score	score
1	Sampling methods	Sampling method (manta trawl), location, materials, date, depth mentioned.	2
2	Sample size	Trawling for >500 m, volume ≈ 1200 m3	2
3	Sample processing and storage	Rinsed with unfiltered water, sieved in the field (acceptable given the large sample volume), precautions were taken to prevent contamination	2
4	Lab preparation	Cotton lab coat, surfaces wiped, as much as possible.	1
5	Clean air conditions	Not applicable [a)]	2
6	Negative controls	Not applicable [a)]	2
7	Positive controls	Not applicable [a)]	2
8	Sample treatment (surface water)	Not applicable [b)]	2
9	Polymer ID	> 1.5 mm: all particles, with automated Near Infrared Spectroscopy (NIR)<br><br>1 – 1.5 mm: all particles up to a maximum of 50 particles per sample, manually with Fourier Transform Infrared Spectroscopy with Attenuated Total Reflection (ATR-FTIR)	2
Total			17

[a)] If a criterion is not applicable to an aspect of the method a maximum score of 2 is assigned (Koelmans et al, 2019). Given the expected absence of contamination and particles losses for large particles > 1 mm, this rule was applied.

[b)] If no chemical digestion of the sample is required, the criterion is deemed no applicable and a maximum score of 2 is assigned.

Table S1. Sampling circumstances & overall count

S	1	2	3	4	5	6	7	8	9	10
Current	BENG	BENG	BENG	BENG	BENG	BENG	SA-SEC	SA-SEC	SA-SEC	SA-SEC
Boat sp	2.4	2.9	4	4	5.2	5.5	5.7	4.9	4.6	4
Wind sp	7.3	10	14		13	18	16.9	17	16	16
Sea st.	Slight	Slight	Mod	Mod	Rough	Rough	Mod	Rough	Mod	Slight
First count	14	10	46	17	21	14	5	1	7	5

S	11	12	13	14	15	16	17	18	19	20
Current	SA-SECC	NECC	NECC	NECC	NECC	NEC	NEC	NEC	NEC	CNA
Boat sp	4.4	4.1	4.5	5	4.8	1.5	4.3	5	4.8	4.4
Wind sp	17	15	10.9	14.9	12.5	Doldrums	4.6	11		15.7
Sea st.	Slight	Slight	Slight	mod	mod	Slight	Slight	Slihgt	Slight	Slight
First count	0	1	2	0	0	0	0	25	9	8

Ns = 20

S	21	22	23	24	25	26	27	28	29	30
Current	CNA	CNA	CNA	CNA	CNA	CNA	CNA	CNA	CNA	CNA
Boat sp	4.4	4.1	2.5	4.2	4.3	4.5	4.3	3.6	4.3	2.3
Wind sp	15.7	9.9	6.4	3.6	10.2	8.4	15.5	14	16	6.5
Sea st.	Slight	Slight	Smooth	Smooth	Slight	Mod	Mod	Choppy	Smooth	slight
First count	140	318	783	1559	1730	135	320	226	416	131

S	31	32	33	34	35	36	37	38	39	40
Current	CNA	CNA	CNA	CNA	NAC	NAC	NAC	NS	NS	NS
Boat sp	3.4	3.5	3.4	3.1	2.6	3.3	3.7	3.1	3.4	3.4
Wind sp	5.9	18.7	11.3	7.7	6.9	5.9	12.1	8.6	4.3	slight
Sea st.	Smooth	Mod	Slight	Mod	Slight	Slight	Mod	Slight	Slight	slight
First count	205	153	115	166	41	32	7	52	40	65

Table S1: boat speed, wind speed, sea state and count for each sample. Samples 20-40 were selected for further laboratory analysis, seeing their overall higher abundancies, calmer seas and consistent reliable boat speeds (not exceeding 5 knots).

Legend:
BENG = benguela current
SA-SEC = south atlantic equatorial current
SA-SECC = south atlantic equatorial counter current
NECC = north equatorial counter current
NEC = north equatorial current
CNA = centre north atlantic
NAC = north atlantic current
NS = north sea

Table S2: Overview of abundancies and frequencies of found categories per polymer type

	Total (%)	Tot (N)	Fragment (%)	Frag. (N)	Foam (%)	Foam (N)	Film (%)	Film (N)	Pellet (%)	Pellet (N)	Line (%)	Line (N)
PE	88	4299	71.9	3484	0	0	9.8	472	0.3	13	6.8	331
PP	10.5	508	7.7	372	0	0	0.5	23	0	0	2.3	113
Other	0.7	34	0.4	20	0.1	3	0.1	3	0	1	0.1	7
Total (N = 4841)	100	4841	80	3875	0.1	3	10.3	498	0.2	14	9.3	451

Table S3: Overview of abundancies and frequencies of shape categories

	Frequency	Percent (row)
Fragment	5586	80.5
Film	853	12.3
Line	484	7.0
Pellet	15	0.2
Foam	4	0.1
Total	9642	100
<5mm (microplastics	5689	81.9
>5mm macroplastics	1253	18.1

Table S4: Variety of findings for polymer type category ‘other’

	Freqency	Type	Class
*calcium carbonate	2		NA
*cellophane	1		NA
*PE	24		PE
*PEP	8	Polyolefin	Other
*PET	1	PET	Other
*polyolefin/PP	4	Polyolefin	Other
*PP	25		PP
*PP	1		PP
*PP+PE	4	Polyolefin	Other
*PS	2	PS	Other
*PVA	1	PVA	Other
*undefined polymer	2		NA
*undetermined	7		NA
*undetermined polymer	16		NA
*zein	5		NA
na	6		NA
NIR	41		NA
PEP	1	Polyolefin	Other
PP+PE	1	Polyolefin	Other
PS	13	PS	Other
PVC	2	PVC	Other
undetermined	68		NA
NA	0		NA

*Polymer type with a * refer to a match <80% with this polymer type. Using expert knowledge, these spectra were examined and added to their respective polymer groups when matching the reference database.*

Table S5: Polymer type libraries

**libraries**
HR Hummel Polymer and Additives
HR Polymer Additives and Plasticizers
HR Specta Polymers and Plasticsizers by ATR
Hummel Polymer Sample Library
Organics by Raman Sample Library
Sigma Biological Sample library

Polymer type libraries used. (software: OMNIC Picta, Thermo Fisher Scientific. see: https://www.thermofisher.com/order/catalog/product/INQSOF018#/INQSOF018)

Table S6: Length and width measurements using the manual measurement tool versus Ferrets diameter and bounding rectangle measurements

particle	W measurement manual 10x										Mean (µm)
1	1030	1232	1181	1232	1075	1130	1044	1213	1023	1232	1139
2	854	854	854	854	854	854	854	854	854	854	854
3	1730	1589	1467	1505	1523	1505	1486	1476	1429	1505	1522
4	1186	1163	1163	1186	1102	1140	1054	1078	1181	1186	1144
5	997	997	997	997	997	997	997	997	997	997	997
6	1008	1008	1094	1017	1017	0.959	1081	0.921	1034	1017	827.8
	L measurement manual 10x										Mean (µm)
1	1491	1543	1442	1250	1318	1145	1232	1302	1237	1344	1330
2	993	1043	1211	1291	1098	905	1179	1072	1127	1181	1110
3	2087	2329	2109	2120	2183	2019	2172	2068	2078	2002	2117
4	2179	1884	2023	2204	2055	1907	2075	1864	1951	1839	1998
5	1365	1326	1214	1214	1232	1248	1241	1278	1263	1140	1252
6	1216	1216	1278	0.993	1275	1075	1017	1337	1181	1116	1071

measurement per particle using the manual measurement tool in ImageJ. Most realistic length and width measurement is established by measuring particles 10 times, to reduce the influence of subjectivity, and mean values are calculated.

particle	Length (µm)	Width (µm)	Ferrets L (µm)	Ferrets W (µm)	BR L (µm)	BR W (µm)	W - F W (mm)	W - BR W (mm)	L – FL (mm)	L - BR L (mm)
1	1330	1139	1271	1086	1163	1124	0.053	0.015	0.0594	0.167
2	1110	854	1166	882	1085	1047	-0.028	-0.193	-0.0560	0.0250
3	2117	1522	2194	1351	2093	1357	0.171	0.165	-0.0773	0.0237
4	1998	1144	1961	1023	1744	1357	0.121	-0.213	0.0371	0.254
5	1252	997	1271	1004	1047	1085	-0.007	-0.088	-0.0189	0.205
6	1071	828	1189	834	1124	853	-0.006	-0.025	-0.118	-0.0528

ferrets diameter measurements (Ferrets W, Ferrets L) and bounding rectangle measurements (BR L, BR W). The difference is calculated between values for Ferets diameter and Bounding Rectangle with manually measured width (W) and length (L). Ferets diameter showed an overall lower difference.

Table S7. Line classification

Model	Classes	LL	BIC	Entropy	p_{min}	p_{max}	n_{min}	n_{max}	p_{BLRT}
	1	-2058	4128	1	1	1	1	1	
	2	-1925	3881	0.610	0.821	0.963	0.40289	0.59711	0
	3	-1897	3843	0.639	0.715	0.949	0.13636	0.50000	0
	4	-1893	3854	0.594	0.578	0.914	0.13017	0.39876	0.4286
	5	-1888	3862	0.651	0.610	0.892	0.03512	0.36570	0.2500

Table S8. Film classification

Model	Classes	LL	BIC	Entropy	p_{min}	p_{max}	n_{min}	n_{max}	p_{BLRT}
Free M, S2	1	-3279	6591	1	1	1	1	1	
Free M, S2	2	-2661	5390	0.906	0.900	0.992	0.16295	0.83705	0
Free M, S2	3	-2526	5153	0.806	0.833	0.960	0.13716	0.58148	0
Free M, S2	4	-2429	4994	0.817	0.826	0.948	0.03986	0.52403	0
Free M, S2	5	-2389	4947	0.836	0.874	0.939	0.03986	0.34232	0
Free M, S2, r	1	-3279	6591	1	1	1	1	1	
Free M, S2, r	2	-2581	5237	0.794	0.855	0.980	0.231	0.769	0
Free M, S2, r	3	-2445	5005	0.725	0.820	0.944	0.144	0.505	0
Free M, S2, r	4	-2387	4930	0.732	0.797	0.923	0.122	0.404	0
Free M, S2, r	5	-2352	4900	0.755	0.796	0.924	0.0422	0.362	0

Table S9: Fragment classification

Model	Classes	LL	BIC	Entropy	p_{min}	p_{max}	n_{min}	n_{max}	p_{BLRT}
Free M, S2	1	-16555	33153	1	1	1	1	1	
Free M, S2	2	-12490	25066	0.848	0.892	0.981	0.226	0.774	0
Free M, S2	3	-11503	23136	0.871	0.752	0.977	0.0473	0.697	0
Free M, S2	4	-11012	22197	0.861	0.722	0.966	0.00714	0.604	0
Free M, S2	5	-10766	21747	0.809	0.733	0.930	0.00535	0.446	0
Free M, S2, r	1	-16555	33153	1	1	1	1	1	
Free M, S2, r	2	-12434	24962	0.820	0.883	0.980	0.250	0.750	0
Free M, S2, r	3	-11098	22343	0.810	0.701	0.962	0.0248	0.596	0
Free M, S2, r	4	-10704	21606	0.741	0.713	0.914	0.0146	0.364	0
Free M, S2, r	5	-10472	21195	0.759	0.683	0.913	0.00196	0.350	

A. Fragments

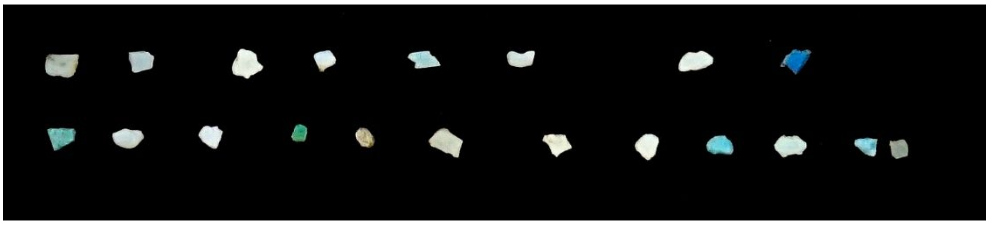

B. Film

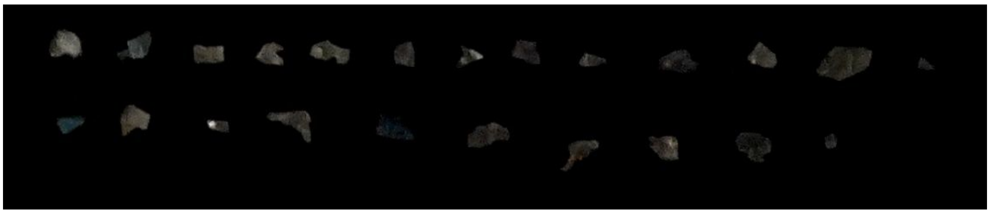

C. Lines

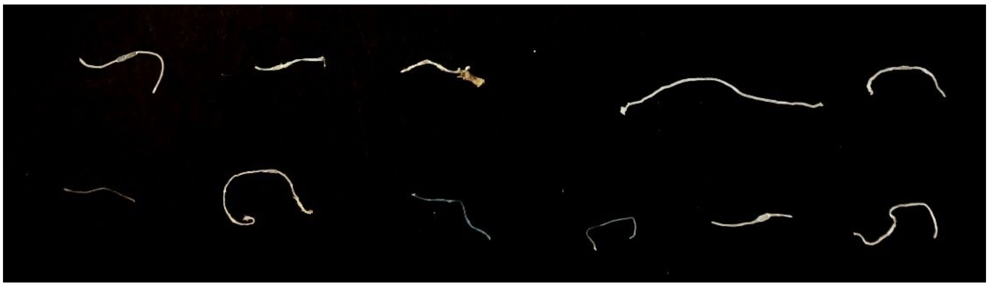

Figure S1. Typical examples of the shape categories fragments (A), film (B) and lines (C).

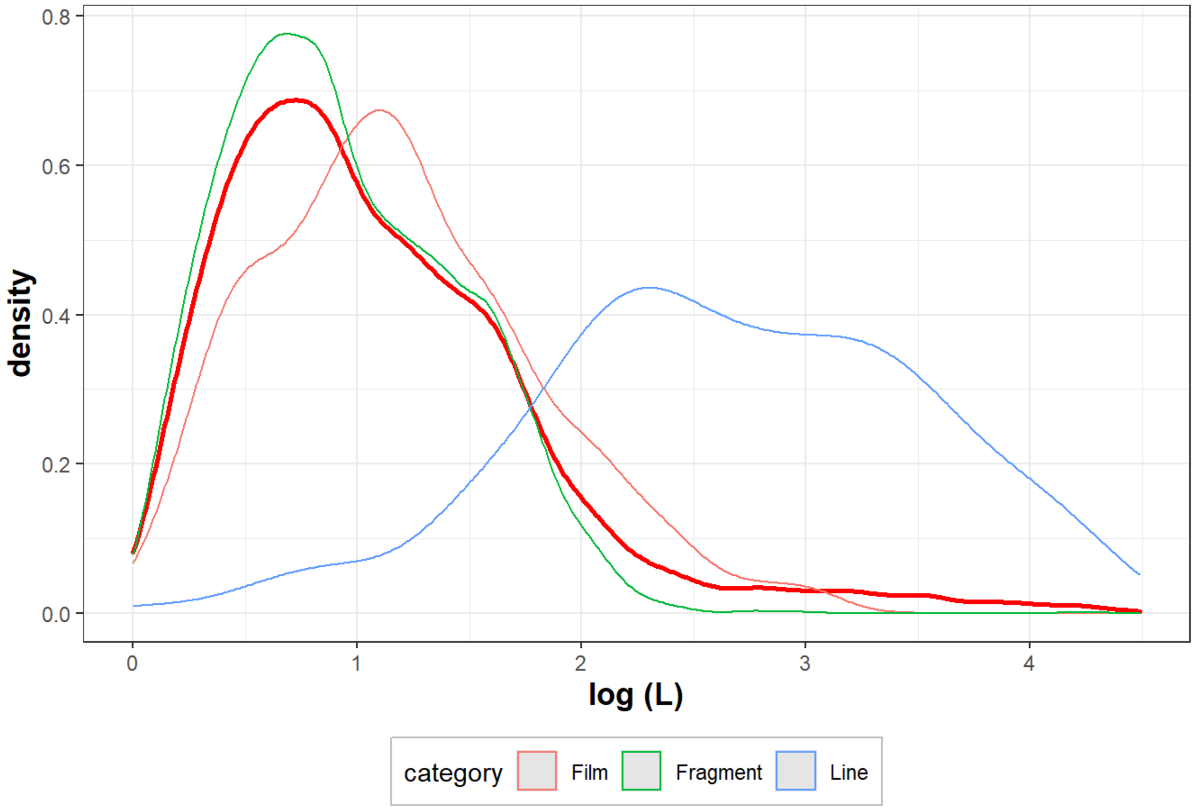

Figure S2: Density distribution for particle length (L, mm) for film, fragment and line. The red line shows the total distribution of all particles together.

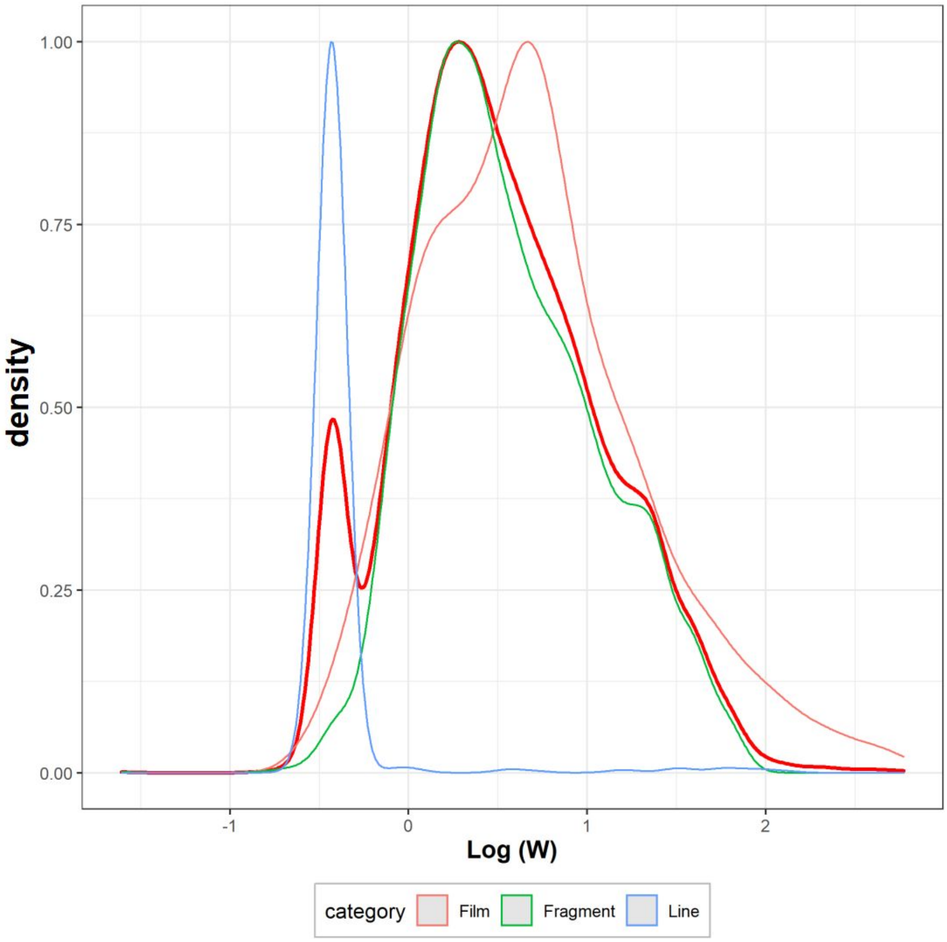

Figure S3: Density distribution for particle width (mm). The red line shows the total distribution of all particles together.

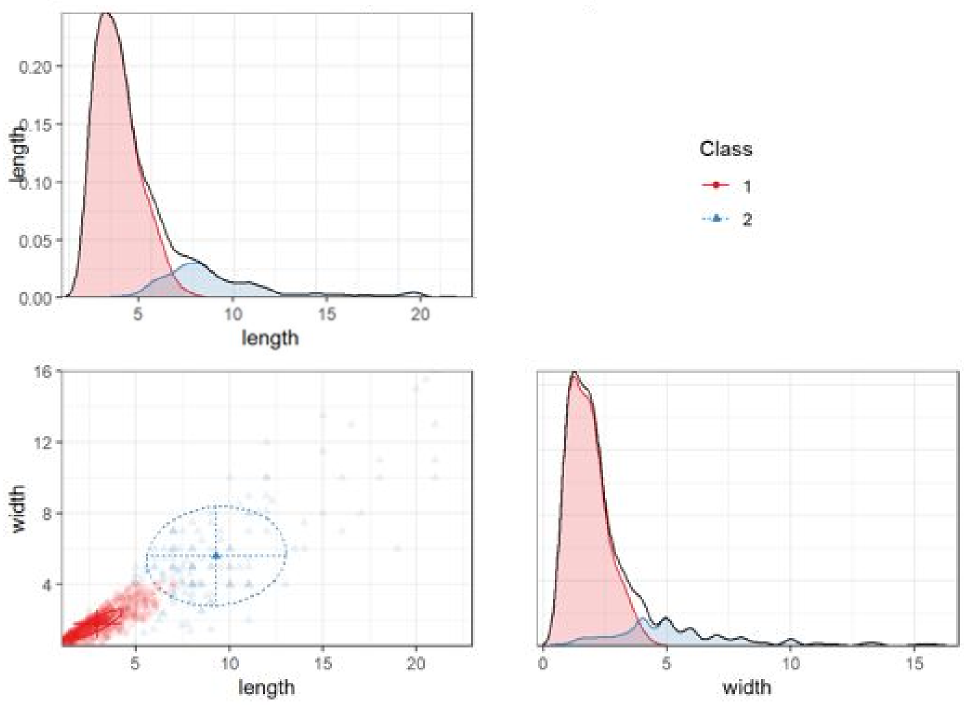

Figure S4. Mixture model of film (2 class solution) with free means and variances, and fixed covariances. Length and width in mm.

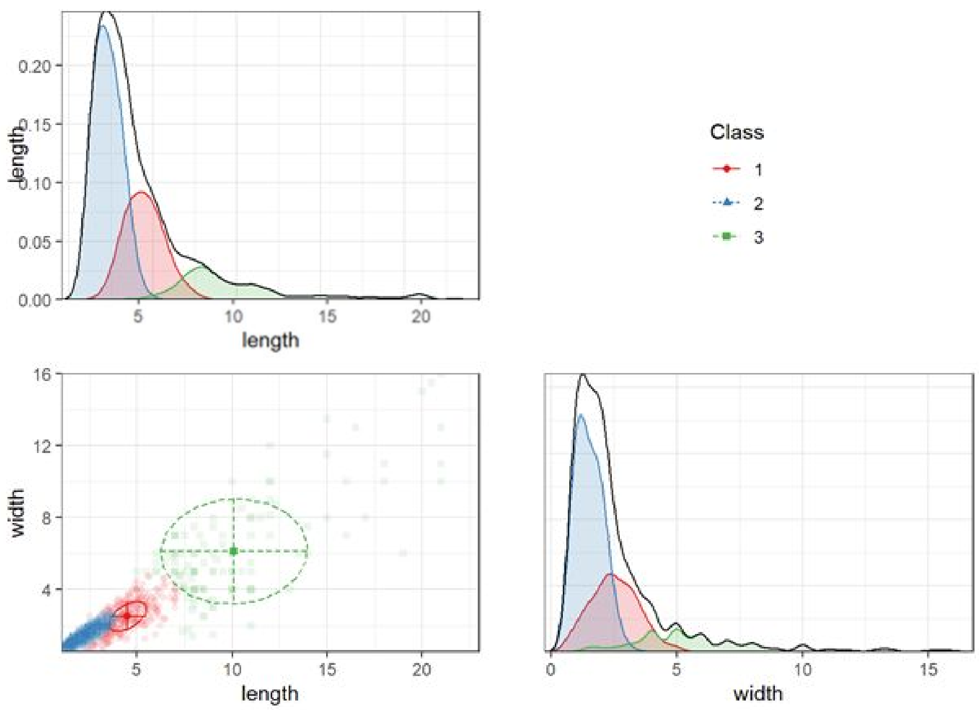

Figure S5. Mixture model of film (3 class solution) with free means and variances, and fixed covariances. Length and width in mm.

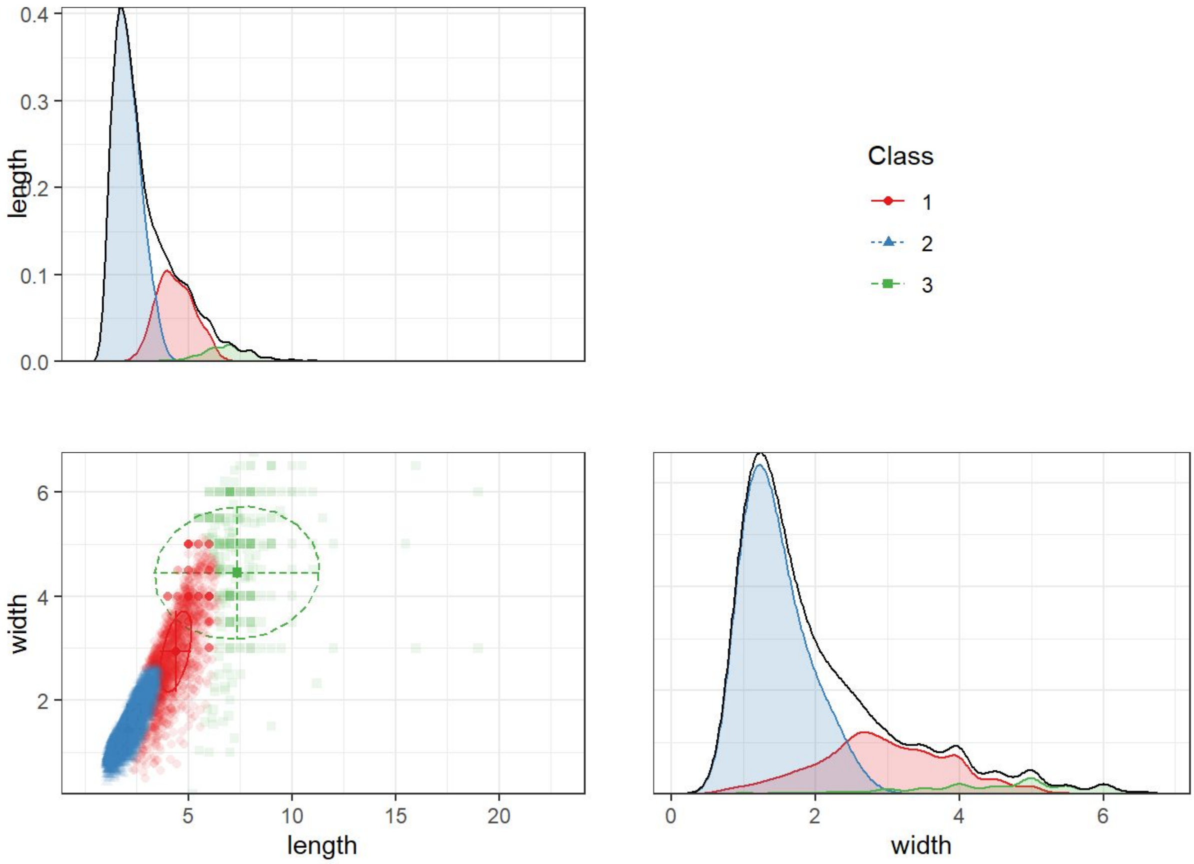

Figure S6. Mixture model of fragments (3 class solution) with free means and variances, and fixed covariances. Length and width in mm.
